# What determines the income gap between French male and female GPs - the role of medical practices

**DOI:** 10.1186/1471-2296-13-94

**Published:** 2012-09-21

**Authors:** Magali Dumontet, Marc Le Vaillant, Carine Franc

**Affiliations:** 1 CERMES3, Inserm U988, CNRS, UMR 8211, 7 rue Guy Moquet, 94801, Villejuif Cedex, France; 2 Université Paris-Dauphine, Paris, France

**Keywords:** Family doctors, General practitioners, Income, Gender differences, Oaxaca-ransom decomposition

## Abstract

**Background:**

In many OECD countries, the gender differences in physicians’ pay favour male doctors. Due to the feminisation of the doctor profession, it is essential to measure this income gap in the French context of Fee-for-service payment (FFS) and then to precisely identify its determinants. The objective of this study is to measure and analyse the 2008 income gap between males and females general practitioners (GPs). This paper focuses on the role of gender medical practices differentials among GPs working in private practice in the southwest region of France.

**Methods:**

Using data from 339 private-practice GPs, we measured an average gender income gap of approximately 26% in favour of men. Using the decomposition method, we examined the factors that could explain gender disparities in income.

**Results:**

The analysis showed that 73% of the income gap can be explained by the average differences in doctors’ characteristics; for example, 61% of the gender income gap is explained by the gender differences in workload, i.e., number of consultations and visits, which is on average significantly lower for female GPs than for male GPs. Furthermore, the decomposition method allowed us to highlight the differences in the marginal returns of doctors’ characteristics and variables contributing to income, such as GP workload; we found that female GPs have a higher marginal return in terms of earnings when performing an additional medical service.

**Conclusions:**

The findings of this study help to understand the determinants of the income gap between male and female GPs. Even though workload is clearly an essential determinant of income, FFS does not reduce the gender income gap, and there is an imperfect relationship between the provision of medical services and income. In the context of feminisation, it appears that female GPs receive a lower income but attain higher marginal returns when performing an additional consultation.

## Background

In France, as in many OECD countries, there has been a recent and strong feminisation of medicine, particularly for general practitioners (GPs) [1,2]. In 2009, 29% of GPs were women vs. 13% in 1983 (source: Eco-Santé), and more than two thirds of medical students who chose general practice were women. Numerous studies showed that women GPs typically work less than men (both number of days per week and number of weeks per year) [3,4], which may result in short-term potential problems in terms of both access to ambulatory care and general practice regulation. It is therefore essential to understand the determinants of GPs provision in terms of quantities and services provided and thus the determinants of the income gap of GPs.

There have been many studies on the measurement and analysis of the income gap between men and women in the field of labour economics [5,6], and these studies always show an income gap in favour of men regardless of country [7,8], business sector [8,9], status (self-employed or salaried) [10] and executive or non-executive status [11]. In the context of health, the first studies that analysed the income gap between male and female physicians were conducted in the United States during the 1970s [12,13]. Kehrer and Langwell (1976, 1982) found differentials of approximately 30% and 22%, respectively, in hourly income in favour of men. Less than one third of each of these differentials is explained by individual doctors’ characteristics (28% and 13%), such as experience, status (salaried or private practitioner) and medical specialty. More recently, Baker (1996) [14] also highlighted an income gap of 41% in favour of men and an hourly wage gap of 14%. After controlling for speciality and practice setting, Baker was the first to show that female GPs had a 13% higher hourly income. Using the same physician dataset, Bashaw and Heywood (2001) explained these results by the differences in working time productivities: women typically worked fewer hours than men, and because of the “law” of diminishing marginal productivity, women had higher hourly earnings than men. Thus, the decrease in working time productivity of physicians is according to the authors in favour of women because they worked fewer hours [15].

Only two similar studies were recently conducted in Europe. Theurl and Winner (2011) obtained similar results to those obtained for U.S. doctors: a 32% income gap and a 20% hourly income gap that both were in favour of Austrian male doctors [16]. Gravelle et al. (2010) measured the income and hourly income gaps between male and female GPs in the United Kingdom of approximately the same magnitude (30% for income and 11% for hourly income). Again, the gaps are in favour of male GPs. The differences in doctors’ characteristics were found to explain between 35% and 66% of the income gap, and interestingly, the marginal return of one working hour is greater for women than that of men [17].

To date, no comparable study has been conducted in France. Dormont and Samson (2008) measured the income gap between male and female French GPs at 34% in favour of men, accounting for experience, type of practice, duration of work, region of practice and the density of doctors and specialists but failing to adequately analyse the determinants of the gap [18]. In France, a fee-for-service payment prevails to compensate private medical practices. This system remains tightly regulated by health insurance: there are annual negotiations between national health insurance and physicians’ unions, where a convention fixes the costs of services (e.g., the cost of a consultation, visit, or specialised procedure). Only doctors belonging to “Sector 2” have the option of charging extra fees (representing only 12% of French GPs in 2009, source: Eco-Santé). GPs may also perform varying activities as a doctor in a private practice and being an employee (hospital, rest home). In this context, the income from a private practice activity should be linked to the number and type of services performed. This mechanism, in theory, should reduce the income gap between male and female GPs, or at least address the gap associated with the choice of activity.

In this study, we measured the income gap between male and female GPs working in the southwest region of France, and we thoroughly analysed its determinants to better identify potential differences and consequences in choices of medical practice and care delivery between men and women doctors.

## Methods

### Data

We derived our sample from a study conducted among private GPs in the southwest region of France (Midi-Pyrénées). The study, in collaboration with the Regional Association of Private Medical Practitioners (URML), was conducted by mail between April and July of 2010. A letter announcing the study and detailing its objectives was sent to all GPs practicing in the region two weeks prior to the delivery of the anonymous questionnaire. The URML report included approximately 3,000 GPs, thereby representing the entire population of GPs in the region. The questionnaire was designed to be completed in 20 minutes and required the GPs to use fiscal documents (Additional file 1). Each responding GP received compensation. This compensation may introduce a selection bias; however, it will be seen that our sample is representative of French GPs. Primarily due to budgetary constraints, we decided to close the inclusion period upon receiving the first 450 questionnaires and no later than four months after the beginning of the study. A follow-up letter was sent within three weeks subsequent to the delivery of the questionnaire. After four months, we had collected 423 usable questionnaires (over 438 received). Among these questionnaires, 84 GPs (19.9%) did not report their turnover in 2008. According to our study, we had to exclude these GPs, which resulted in a final sample of 339 GPs. Table 1 shows that there was no statistically significant difference between the two groups of GPs.

**Table 1:** Differences between turnover respondents and non-respondents

**Variables**	**Turnover respondents N = 339**	**Turnover non-respondents N = 84**	**Test ‘**
Age	52.1	52.15	NS
Gender	0.72	0.71	NS
Rural region	0.4	0.33	NS
Years of experience (Number of years in private practice)	21.3	21.7	NS

*‘ T-test*.

Our sample is representative of French GPs; the average age in our sample (n = 339) is the same as that for all French GPs (52 years old) as is the proportion of female GPs (29%, source: Eco-Santé). The sample is also representative in terms of income: the average income of private medical practitioners (net of charges and social security contributions) in our sample is 71,364 Euros, while the average is 71,690 Euros for all French GPs [19].

Two income variables were designed such that

• The gross profit of private medical practice (GPPMP) is such that

(1) $$\text{GPPMP}=(\text{private medical practice turnover}–\text{fees for a locum})\times \left(1–\text{average operating expense rate}\right)$$

The private medical practice turnover and fees for a locum were collected in the study. The doctors used data from official documents (Système National Inter-Régimes (SNIR) data for the turnover and declaration number 2035A / tax return). The average operating expense rate was estimated by the French ministry of Health as 25.3%. This rate excludes taxes and social security contributions and corresponds, on average, to personnel expenses, acquisition, rent, and transportation fees [20].

• We defined the gross income (GI) based on the variable GPPMP,

(2) GI=GPPMP+Salaries+Indemnities for teaching and training+Residency indemnities+Other benefits

The salaried activities and various indemnities are exclusively declarative data (self reported). GI represents the gross income of doctors (before paying income tax). We used this variable in our study.

### Data analysis

Firstly, we specified an econometric model (least-squares model) to identify the main determinants of income and four groups of variables: GPs’ characteristics, workload, type of practice and type of patients (Additional file 2). Due to the sample size, we decided to be parsimonious and not include many explanatory variables. Using the backward method, we chose the most significant explanatory variables, which can be grouped in three categories: GPs’ characteristics, workload and type of practice.

Using these explanatory variables, three models were specified: one for all GPs and one for each of the two subgroups (male, female) (see Appendix A). To study the determinants of the income gap between males and females, we used the Oaxaca-Ransom decomposition (1994) [21], and thus, we used the entire GP population as a reference group; this technique enabled us to distinguish the part of the income gap due to the differences in the distribution of explanatory variables (of income) between women and men, denoted the “explained part” from the part due to differences in the effects (returns) of these variables on income, denoted the “unexplained part”. This last part, the unexplained part, can be divided into two parts: a male advantage when the effect of an explanatory variable (years of experience, location, etc.) on male income is greater than the effect of the same variable on income of the reference population and a female disadvantage when the effect of a variable on female income is greater than its effect on the income for all GPs (see Appendix A). We checked the robustness of our results with three different income variables (private practice turnover, GPPMP, GI), and because the results were very similar, we chose to only present the results of the GI variable (others results are available upon request).

## Results

### Male/female differences

The descriptive statistics are presented in Table 2. GI is on average 80,788 Euros for females and 109,048 Euros for males, corresponding to a gap of 26% (using the male income as the reference). This income gap partially reflects the gender differences in workload: for example, women were found to perform, on average, 33% fewer services than men (number of consultations and visits). Moreover, women worked significantly less in terms of days worked per week and hours worked per working day and reported more vacation days than men. Gender differences in workload appeared to be larger than the income gap.

**Table 2:** Descriptive Statistics

***Variables***	***N***	***Mean***	***N***	***Male***	***N***	***Female***	***T-Test***
Gross Income (GI)	327	101097,43	235	109048,23	92	80788,34	***
		[46539,44]		[42272,94]		[50831,52]	
***GPs’ Characteristics***							
Age	327	52.05	235	53.22	92	49.07	***
		[7.54]		[7.08]		[7.88]	
Sector 2 (Unregulated fees)	327	0.06	235	0.06	92	0.07	NS
Rural region	327	0.34		0.36	92	0.27	NS
Having a child under the age of 15	333	0.36	241	0.33	92	0.45	*
Teaching position	335	0.21	239	0.22	96	0.16	NS
Reported being in good health	335	0.91	240	0.92	95	0.89	NS
Years of experience	334	21.34	239	23.01	95	17.12	**
		[9.109]		[8.40]		[9.49]	
***Intensity of medical activity***							
Number of consultations/visits per year	327	4674.74	235	5150.62	92	3459.17	***
		[2149.51]		[2148.51]		[1613.55]	
Salaried activity (%)	327	0.10	235	0.11	92	0.1	NS
		[0.21]		[0.21]		[0.22]	
Number of hours worked per day	324	10.95	234	11.09	90	10.56	*
		[2.15]		[2.23]		[1.88]	
Number of working days per week	337	4.63	231	4.81	91	4.26	***
		[0.88]		[0.76]		[1.01]	
Number of vacations (weeks)	333	5.57	241	5.37	92	6.08	*
		[2.43]		[2.38]		[2.52]	
***Type of practice***							
Group practice	327	0.46	235	0.45	92	0.48	NS
Participating in on-going care	324	0.69	233	0.73	91	0.62	**
Specialised practice	314	0.15	230	0.15	84	0.17	NS
Percentage of visits	330	13.73	238	15.17	92	10.1	**
		[10.22]		[10.73]		[7.66]	
Have consultations by appointment	339	0.39	243	0.35	96	0.49	*
Frequently perform gynaecologic follow-ups	331	0.34	237	0.24	94	0.60	***
Frequently perform obstetric follow-ups	324	0.26	233	0.21	91	0.41	***
Frequently perform electrocardiograms	300	0.19	221	0.23	79	0,07	***
Frequently perform paediatric follow-ups	334	0.81	240	0.78	94	0.89	**
Frequently perform minor surgeries	334	0.25	240	0.28	94	0.16	**
Frequently perform traumatology	331	0.35	238	0.38	93	0.27	*
***Type of patients***							
Have many children in their patient list (aged under 16)	324	0.52	232	0.47	92	0.62	**
Have many elderly people in their patient list (aged over 65)	326	0.43	233	0.47	93	0.33	**

***Notes***: ***, ** and * denote significance at 1, 5 and 10% levels, respectively.[] Standard Error.

Male and female GPs also appeared to provide different types of services: for example, 60% of female GPs reported frequently performing gynaecologic follow-ups vs. 24% of male GPs, whereas male GPs reported performing electrocardiograms, minor surgeries, and traumatology more often than female GPs.

Descriptive statistics also show significant differences between male and female GPs with respect to their own characteristics, such as age (p < 0.01) and experience (p < 0.05), and with respect to their workload in terms of number of medical services (consultations and visits, p < 0.01), number of days worked per week (p < 0.01), percentage of visits (p < 0.05) and the percentage of children in their patient list (p < 0.05).

### Regression results

The ordinary least-squares estimates of GI are reported in Table 3: the first column indicates the estimates for the income of all GPs regardless of gender; the next two columns present the respective results of estimates for men and women.

**Table 3:** Income estimates

**Variables**	**Pooled**		**Male**		**Female**	
	Coef.		Coef.		Coef.	
Constant	10.1454	***	10.5155	***	9.7848	***
***GPs’ characteristics***						
Years of experience	0.0293	***	0.0114	**	0.0591	***
Years of experience squared	−0.0006	***	−0.0002	NS	−0.0017	***
Sector 2 (Unregulated fees)	0.0475	NS	−0.0353	NS	0.2702	**
Sector 1 (Regulated fees)	Ref.		Ref.		Ref.	
***Location***						
Rural regions	0.1210	***	0.0980	***	0.1776	***
Urban areas	Ref.		Ref.		Ref.	
***Workload***						
Number of consultations/visits per year	0.0002	***	0.0001	***	0.0002	***
Salaried activity (%)	0.7685	***	0.6900	***	0.8369	***
***Type of practice***						
Group practice	0.0575	*	0.0826	***	−0.0032	NS
Solo practice	Ref.		Ref.		Ref.	
N	321		233		88	
F-test	100.78***		57.47***		55.22***	
Adjusted R^2^	0.73		0.68		0.84	

***Notes***: ***, ** and * denote significance at 1, 5 and 10% levels, respectively.

The Chow test [22] indicated that the null hypothesis of equality of all coefficients in the male and female incomes models can be rejected at the 5-percent level, indicating that the income structures for male and female GPs are different. Considering all GPs, we can show that the years of experience (number of years in private practice) has a significant, positive effect (p < 0.01) on the GI that decreases over time (p < 0.01). GI increases during the first years of a GP’s career and then stabilises and decreases on average from the 24^th^ year of experience. This well-known result could be due to an age effect and/or a generation effect. This result is in accordance with previous studies [18]. Moreover, we found that GPs practicing in rural regions have a greater income than those practicing in urban areas (p < 0.01) [23], which could be explained by different patients profiles and/or by the type of services provided. The fee-for-service payment theoretically implies a direct relationship between private practice income and the number of services performed. As expected, the number of services performed by GPs had a significant, positive effect on GI (p < 0.01): an increase in the number of services by 100 results in an increase in the GI by 2%. Salaried activity appears to have a positive effect on income (p < 0.01). GP group practices also had a positive effect on GI; however, this effect has a low significance (p < 0.10). Finally, GPs in ‘Sector 2’ were allowed to charge extra fees; however, this had no significant effect on their GI, noting that we controlled for the number of medical services provided by the GP [24,25].

Comparing the estimates of the explanatory variables effects with the income of male and female GPs, we discovered several differences: for example, considering the years of experience, an additional year in private practice had a positive effect for all GPs; however, this effect is greater on the income of women than of men. We also observed that with all other factors being equal, women had a higher GI when they offered an additional consultation or visit, which could be due to the type of service offered. Similarly, the impact of a salaried activity in addition to private practice was positive for both male and female GPs but greater for female GPs than for male GPs.

### Decomposition of the gross income (GI) gap

The results of the decomposition are presented in Table 4: the total GI gap between male and female GPs was estimated to be 0.453. This total gap corresponds to the sum of three parts: one part is explained by the average differences in characteristics, denoted the explained part; a second part, denoted the unexplained part, corresponds to the sum of the advantages and disadvantages of male and female GPs (compared to the entire GP population) for each explanatory variables; and finally, the third part is explained by the differential between the constants estimated for the two models.

**Table 4:** Decomposition of the difference in income between male and female GPs

**Explanatory Factor**	**Male Advantage **$\sum_{k=1}^{n}\bar{X_{k}^{M\;}}\left({\hat{\beta }}_{k}^{M}-{\hat{\beta }}_{k}^{\mathrm{Ref}}\right)$**1**		**Female Disadvantage **$\sum_{k=1}^{n}\bar{X_{k}^{F\;}}\left({\hat{\beta }}_{k}^{\mathrm{Ref}}-{\hat{\beta }}_{k}^{F}\right)$**2**		**Differences of returns to characteristics = (1) + (2) **		**Endowment term **$\sum_{k=1}^{n}{\hat{\beta }}_{k}^{\mathrm{Ref}}\left(X_{k}^{M}-X_{k}^{M}\right)$	
Experience’	−0.147	*	−0.089		−0.236		0.040	**
Sector 2	−0.005	*	−0.013		−0.017		−0.000	
Rural regions	−0.003		−0.011		−0.014		0.011	
Number of consultations and visits per year	−0.185	***	−0.185	***	−0.369	***	0.275	***
Salaried activity (%)	−0.008	**	−0.007		−0.015		0.004	
Group practice	0.011		0.031		0.041		0.003	
**Subtotal**	**−0.337**	***	**−0.273**	***	**−0.611**	***	**0.333**	***
Constant ${\hat{\beta }}_{0}^{M}-{\hat{\beta }}_{0}^{F}$	0.731							
**Total: (log income difference)**	**0.453**							

*Notes:*

***‘****We gathered the experience effect (years of experience + years of experience squared).*

This decomposition with the reference being the entire GPs population isolated two terms: one for the male advantage relative to the reference and another for the female disadvantage. We summed these two terms and obtained part of the income gap due to differences of the returns to characteristics (for each characteristic)

***, ** and * denote significance at 1, 5 and 10% levels, respectively.

The part explained by the average differences in characteristics is equal to 0.333 (i.e., 73% of the gap 0.333/0.453). It is interesting to note that the variable reflecting GP workload (number of medical services) explains 83% of differences in the characteristics i.e., 61% (0.275/0.453) of the total GI gap between male and female GPs. The difference in years of experience in the private practice explains 8% of the GI gap.

We now study the gender differences in returns of the different explanatory variables on GI: the first term reflects the nature of the returns for men relative to the GP population and corresponds to an advantage for male when the term is positive (Appendix A); the second term reflects the nature of returns for women that corresponds to an disadvantage if the term is positive. Female GPs appear to benefit from a total advantage in their estimated coefficients relative to the reference sample (-0.273); more precisely, women have an advantage in the marginal return of the workload variable (number of medical services) compared with that of the reference sample. In other words, it is more profitable for female practitioners to perform an additional medical service than it is for the total GP population in the sample. In contrast, men have a disadvantage compared with the reference sample (-0.337) that is highly significant for the workload variable (p < 0.01) as well as for the ‘salaried activity’ variable (p < 0.05). Moreover, male GPs have a disadvantage in years of experience and in Sector 2; however, these differences of returns are only weakly significant.

Finally, the constant differential is the third term of the decomposition is equal to 0.731. This term is difficult to interpret because it gathers the effects of the unobserved differences in returns and differences in the means of unobserved variables and also of the differences in the returns on omitted variables, such as discrimination effects [17].

## Discussion

This paper is the first to examine the nature of the income gap between male and female GPs in France. Women have incomes that are 26% less than those of their male colleagues, which is comparable to the wage gap measured for salaried workers in France, which was 25.3% in favour of men [26]. Our results were also similar to European studies with respect to income for physicians [16,17].

As shown in the literature, the descriptive analysis confirms that the female GPs’ practice differs from male GPs’ practice, notably with respect to workload (e.g., number of hours worked per week, number of working days per week and number of weeks off) [3] and to the number of services provided (consultations and visits) with a 33% differential.

With a fee-for-service payment, we could expect that the number of services provided and the income were strongly associated such that the production of services should explain a large part of the income gap. Using the Oaxaca-Ransom decomposition (1994), we showed that less than three quarters (73%) of the income gap was actually explained by the average differences in the characteristics between male and female GPs and that less than two thirds (61%) of the income gap can be explained by the difference in the number of services provided. The impact of the differences in medical services provision between male and female GPs (33%) is thus far from fully explaining (less than two thirds, 61%) of the gender income gap (26%).

Considering the unexplained part of the income gap, the decomposition also allowed us to highlight the differences in the marginal returns to characteristics of GPs. Similarly to Kehrer, Langwell and Gravelle et al. (1976, 1982, 2010), we showed that female GPs have higher marginal returns that decrease the income gap between men and women by partially compensating for the differences in workload [12,13,17]. In particular, women have a higher marginal return relative to the average of the sample (which is correspondingly lower for men) when they perform an additional service. In the context of the FFS system, this result may be due to the different types of consultations and/or to the different patients’ profiles, implying the provision of specialised procedures paid for in addition to the fee. According to our data, female GPs reported to perform more often than male GPs paediatric follow-ups and gynaecological follow-ups, which imply additional payments in the French system (e.g., five Euros for follow-ups with infants less than two years old corresponding to an extra fee of nearly 25%). These different types of consultations could explain at least partially the difference in marginal returns of a service provided between male and female GPs. Another additional explanation may be derived from the induced demand theory: Delattre and Dormont (2008) showed that facing an increase in medical density and thus being subject to rationing, doctors tend to compensate by increasing the volume of treatments performed per consultation or number of visits [27]. Our results may also be interpreted by following a similar intuition: female GPs who appear to choose to work less or undergo a professional time constraint compensate for this by increasing their workload either by intensifying the type of their consultations and/or by favouring patients and consultations that incur additional payments. Nevertheless, it is rather well-known that female doctors generally see more women and children [28], which can also result from patient demand and preferences.

It is important to note that by controlling for the number of medical services provided, the GP’s gross income can be interpreted as the productivity of the GP (income for a given production). For example, all things being equal, providing an additional medical service for a female GP generates a higher additional income, suggesting a higher productivity. Another important dimension of GP productivity is the length of medical services provided: several studies agreed that female practitioners have longer consultations/visits than male practitioners [24-29]. By considering the length of a consultation/visit as a proxy of quality [30,31], a higher marginal return for women may be viewed as a compensation for quality. Following this line, we could have studied the hourly income gap between male and female GPs; however, in France, physicians are not paid hourly, and thus studying the hourly income gap was not appropriate [15].

The differential between the estimates of constants in the two models finally explains a large part of the income gap. This differential may be derived from unobservable characteristics (preferences, risk aversion, etc.) in two possible ways. First, it could be the result of either the differences in unobservable characteristics. For example, Rizzo (2007) showed that male and female physicians appeared to reveal different ‘target incomes’ [32]. Second, it could be the result of differences in the estimated coefficients of these unobservable characteristics, such as discrimination effects (i.e., patients in rural areas or older patients that are less likely to see a female GP).

We used declarative data for this reason, and therefore, there is a self-reporting bias. However, there is no reason to believe that this bias is different for the two subgroups. Considering the selecting variables, we have chosen to select the significant variables that all were in accordance with the literature. In addition, we did not consider the simultaneity of GPs’ decisions between workload (number of services performed) and income: this could generate an endogeneity bias. We tried to correct this bias with instrumental variables, but we did not find valid instruments in our data. However, Fortin et al. (2010) showed that because the bias is the same for male and female GPs, such decomposition results remain valid [33].

## Conclusions

The findings of this study are helpful for understanding the determinants of income gap between male and female GPs. As is well-known, female GPs work less than male GPs; however, this difference appears to only explain 61% of the total income gap. Even if workload is clearly an essential determinant of income and thus, a determinant of the gender incomes gap, our results contradict our assumption because FFS does not reduce the gender incomes gap, where there is an imperfect relationship between the provision of medical services and income. More interesting in the context of feminisation, female GPs receive a lower income but demonstrate a higher marginal return when performing an additional consultation. To consolidate our results, it would be interesting to use a larger sample and to take into account the life partner’s income. Moreover, a complementary approach could be used to perform a similar analysis of other medical specialties and to consider the simultaneity of the two key decisions: workload and income.

## Appendix A

**Oaxaca-Ransom decomposition**We ran three models:Pooled model for all GPs:

(3) $$ln\left(Y_{i}\right)=\beta_{0}+\sum_{k=1}^{K}X_{i,k}\beta_{k}+u_{i}$$

Two-group models (male, female):

(4) $$ln{\left(Y_{i}\right)}^{g}=\beta_{0}^{g}+\sum_{k=1}^{K}X_{i,k}^{g}\beta_{k}^{g}+u_{i}^{g}\;g=M,F$$

In these equations, i indexes the GPs, Y is the GP’s income, X_k_ represents the matrix of observable characteristics used to explain Y (GP’s characteristics, workload, type of practice) and the superscript g = {M, F} denotes gender.The expression could be written as

(5) $$D=\bar{1n\left(Y^{M}\right)}-\bar{1n\left(Y^{F}\right)}=\sum_{k=1}^{n}\bar{X_{k}^{M}}{\hat{\beta }}_{k}^{M}+{\hat{\beta }}_{0}^{M}-\sum_{k=1}^{n}\bar{X_{k}^{F}}{\hat{\beta }}_{k}^{F}-{\hat{\beta }}_{0}^{F}$$

Oaxaca and Ransom [26] proposed a decomposition based on a non-discriminate norm, which decomposes the average income gap as follows:

(6) $$D=\bar{1n\left(Y^{M}\right)}-\bar{1n\left(Y^{F}\right)}=\underset{1}{\underset{︸}{\left({\hat{\beta }}_{0}^{M}-{\hat{\beta }}_{0}^{F}\right)}}+\underset{\text{Differences of return to characteristic}}{\underset{︸}{\begin{array}{c} \left[\sum_{k=1}^{n}X_{k}^{M}\left({\hat{\beta }}_{k}^{M}-{\hat{\beta }}_{k}^{\mathrm{Ref}}\right)\right]\\ 2 \end{array}+\begin{array}{c} \left[\sum_{k=1}^{n}X_{k}^{F}\left({\hat{\beta }}_{k}^{\mathrm{Ref}}-{\hat{\beta }}_{k}^{F}\right)\right]\\ 3 \end{array}}}+\underset{\text{Differences in characteristic, Explained part}}{\underset{︸}{\begin{array}{c} \left[\sum_{k=1}^{n}{\hat{\beta }}_{k}^{\mathrm{Ref}}\left(\bar{X_{k}^{M}}-\bar{X_{k}^{F}}\right)\right]\\ 4 \end{array}}}$$

1. This component measures the part of the income differential that is associated with the gap between constants. Gravelle et al. (2010) highlighted that the difference between the regression constants for males and females can arise from unobserved differences in returns, differences in the means of unobserved variables and differences in the returns on these omitted variables [17].

2. This term is the difference between the estimated coefficients of men relative to the reference (male advantage if >0).

3. This is the difference between the estimated coefficients of women relative to the reference (female disadvantage if >0).

4. This term represents the differences in characteristics between the two groups, which can be interpreted as the part of the income gap that is associated with the differences in observed variables (e.g., personal characteristics, workload, and the doctor’s practice). The term is one of the income differentials explained by the average differences in characteristics.

## Abbreviations

URML: Union régionale des médecins libéraux; GPPMP: Gross profit of private medical practice; GI: Gross income.

## Competing interests

The authors declare that they have no competing interest.

## Authors’ contributions

MD participated in the analysis and in writing the manuscript. MLV helped with the analysis and interpretation of the findings. CF was involved in the project conception, design, participated in the analysis, and drafted the manuscript. MD and CF contributed towards preparing and revising the manuscript for the article. All authors read and approved the final manuscript.

## Pre-publication history

The pre-publication history for this paper can be accessed here:

http://www.biomedcentral.com/1471-2296/13/94/prepub

## Supplementary Material

Additional file 1 Survey questionnaire. Click here for file

Additional file 2 Description of the available variables. Click here for file

## References

[B1] Levinson W Lurie N When most doctors are women: what lies ahead? Ann Intern Med 2004 41 471 474 15381521 10.7326/0003-4819-141-6-200409210-00013

[B2] Fauvet L Les affectations des étudiants en médecine à l’issue des épreuves classantes nationales en 2011 Études et Résultats, French Ministry of Health 2012 802 1 8

[B3] Fivaz C Le Laidier S Une semaine d’activité des généralistes libéraux Points Stat 2001 330 1 8

[B4] Jakoubovitch S Les emplois du temps des médecins généralistes Études et Résultats, French Ministry of Health 2012 797 1 8

[B5] Altonji JG Blank RM Race and gender in the labor Handb of Labor Economics 1999 3 3143 3259

[B6] Cain GC The economic analysis of labor market discrimination: A survey Handb of Labor Economics 1986 1 693 785

[B7] Blau FD Kahn LM Understanding international differences in the gender pay gap J of labor Economics 2003 21 106 139 10.1086/344125

[B8] Arulampalam W Booth AL Bryan ML Is there a glass ceiling over Europe? Exploring the gender pay gap across the wage distribution Industrial and Labor relations Rev 2007 60 163 186

[B9] Metcalf H Rolfe H Employment and earnings in the finance sector. A gender analysis Equality and hum. Rights comm. Res. Rep. series 2009 1 100

[B10] Hundley G Why women earn less than men in self-employment J of labor res 2001 22 817 829 10.1007/s12122-001-1054-3

[B11] Meurs D Ponthieux S Une mesure de la discrimination dans l’écart de salaire entre hommes et femme Economie et Statistique 2000 337 135 158 10.3406/estat.2000.7501

[B12] Kehrer BH Factors affecting the incomes of Men and women physicians: an explanatory analysis J of Hum Resources 1976 11 526 545 10.2307/145430 977939

[B13] Langwell KM Factors affecting the incomes of Men and women physicians: further explorations J of Hum Resources 1982 17 261 275 10.2307/145472 7130708

[B14] Baker LC Differences in earnings between male and female physicians New Engl J of Medicine 1996 334 960 964 10.1056/NEJM199604113341506 8596598

[B15] Bashaw DJ Heywood JS The gender earnings gap for US physicians: has equality been achieved? Labour 2001 15 371 391 10.1111/1467-9914.00169

[B16] Theurl E Winner H The male-female gap in physician earnings: Evidence from a public health insurance system Health Econ 2010 20 1184 1200 20853520 10.1002/hec.1663

[B17] Gravelle H Hole AR Santos R Measuring and testing for gender discrimination in physician pay: English family doctors J of Health Economics 2010 30 660 674 10.1016/j.jhealeco.2011.05.005 21680033

[B18] Dormont B Samson A-L Medical demography and intergenerational inequalities in general practitioners’ earnings Health Econ 2008 17 1037 1055 10.1002/hec.1387 18702097

[B19] Bellamy V Les revenus libéraux des médecins en 2007 et 2008 Études et Résultats, French Ministry of Health 2010 735 1 8

[B20] Bellamy V Fréchou Les revenus libéraux des professionnels de santé Document de travail, série sources et méthodes 2010 French Ministry of Health, DREES, n° 16 1 58

[B21] Oaxaca RL Ransom MR On discrimination and the decomposition of wage differentials Jof Econometrics 1994 61 5 21 10.1016/0304-4076(94)90074-4

[B22] Chow GC Tests of equality between subsets of coefficients in two linear regressions Econometrica 1960 28 591 605 10.2307/1910133

[B23] Morris S Goudie R Sutton M Gravelle H Elliott R Hole A Ma A Sibbald B Skatun D Determinants of general practitioners’ wages in England Health Econ 2011 20 147 160 10.1002/hec.1573 20127746

[B24] Clerc I L’Haridon O Paraponaris A Protopopescu C Ventelou B Fee-for-service payments and consultation length in general practice: a work-leisure trade-off model for French GPs Appl Econ 2012 44 3323 3337 10.1080/00036846.2011.572858

[B25] Ventelou B Paraponaris A Sebbah R Aulagnier M Protopopescu C Gourheux J-C Verger P Un observatoire des pratiques en médecine générale: l’expérience menée en région Provence-Alpes-Côte-d’Azur Rev Française des affaires soc 2005 1 127 160

[B26] Meurs D Ponthieux S L’écart des salaires entre les femmes et les hommes peut-il encore baisser? Economie et Statistique 2006 398 99 129 10.3406/estat.2006.7119

[B27] Delattre E Dormont B Fixed fees and physician-induced demand: A panel data study on French physicians Health Econ 2003 12 741 754 10.1002/hec.823 12950093

[B28] Labarthe G Les consultations et visites des médecins généralistes, un essai de typologie Etudes et Résult 2006 481 1 8

[B29] Breuil-Genier P Goffette C La durée des séances des médecins généralistes Etudes et Résult 2006 481 1 8

[B30] McGuire TG Physician agency In *Handbook of health economics*. Edited by Elsevier Science; 2000:462–536

[B31] Freeman GK Horder JP Howie JGR Hungin AP Hill AP Shah NC Wilson A Evolving general practice consultation in Britain on issues of length and context BMJ 2002 324 880 882 10.1136/bmj.324.7342.880 11950738 PMC101402

[B32] Rizzo JA Zeckhauser RJ Pushing incomes to reference points: why do male doctors earn more? J of Economic Behav and Organization 2007 63 514 536 10.1016/j.jebo.2005.06.007

[B33] Fortin N Lemieux T Firpo S Ashenfelter O, Card D Decomposition methods in economics Handbook of Labor Economics 2010 Amsterdam: Elsevier science 1 102

