# Supplementary material for: What determines the income gap between French male and female GPs - the role of medical practices
Source: BMC Fam Pract. 2012 Sep 21;13:94. doi: 10.1186/1471-2296-13-94 (PMC3515453; doi:10.1186/1471-2296-13-94)
Supplement: Additional file 1 — Survey questionnaire. [file 1471-2296-13-94-S1.pdf]

Identifiant n° [ ]

A - Organisation de votre pratique

1. **Nature de l'exercice** ☐ Libéral ☐ Libéral + salarié hospitalier ☐ Libéral + vacations ou autres (*élu professionnel, pompier, autre*)

2. **Vous exercez en**
   a - ☐ Secteur 1
   b - ☐ Secteur 1 avec droit de dépassements
   c - ☐ Secteur 2

3. **… dans un**
   a - ☐ Cabinet individuel
   b - ☐ Cabinet de groupe de médecins généralistes — Nbre de médecins :………
   c - ☐ Cabinet de groupe de médecins de différentes spécialités — Nbre de médecins :………
   d - ☐ Maison de santé pluridisciplinaire / centre de santé
   e - ☐ Autre, quelle structure : ………………………………………………………………………

4. **Si vous exercez en groupe partagez-vous …**
   A. les dossiers de vos patients avec vos associés ? ☐ Oui ☐ Non
   B. les dépenses professionnelles ? (salariales, locatives, etc.) ☐ Oui ☐ Non
   b- Si oui, quelle est votre part moyenne des dépenses : … …%

5. **Avez-vous informatisé vos dossiers patients ?** ☐ Oui ☐ Non

6. **« Télétransmettez-vous » à l'assurance maladie ?** ☐ Oui ☐ Non

7. Si oui, quelle part des actes que vous réalisez : … … % des consultations … … % des visites

8. **Pour la prise de RDV, avez-vous recours à…**
   A - ☐ un standard téléphonique de prise de RDV
   B - ☐ un(e) secrétaire salarié(e)
   C - ☐ un(e) conjoint(e) « collaborateur »
   D - ☐ vous prenez vous-même vos rendez-vous
   E - ☐ autre ………………………………………………………………………

9. **Quelles ont été vos dépenses professionnelles au niveau individuel en 2008 ?**
   (Déclaration N° 2035 A -2008)

A- Charges salariales (secrétariat, etc.)	[BB]+[BC]	.. .. €
B- Loyer et charges locatives	[BF]	.. .. €
C- Achats	[BA]	.. .. €
D- Frais de transports	[BJ]	.. .. €
E- Autres charges	[BR] – [charges mentionnées ci-dessus]	.. .. €
F- Honoraires rétrocédés (remplaçants)	[AC]	.. .. €

10. **Recevez-vous les patients sur RDV ?**
    ☐ Oui, uniquement ☐ Oui, la plupart du temps ☐ Non, pas souvent ☐ Non, jamais

11. **Recevez-vous les visiteurs médicaux**
    ☐ Oui, environ 1 fois / semaine ☐ Oui, moins régulièrement ☐ Non, pas souvent ☐ Non, jamais

12. **Participez-vous à la Permanence des soins …**
    A. comme médecin régulateur ? *(plateforme téléphonique, Samu, etc.)* ☐ Oui ☐ Non
    B. comme médecin de permanence ? *(astreintes, maison médicale de garde, association)* ☐ Oui ☐ Non
    si oui, ☐ la nuit et /ou ☐ le WE et de façon ☐ ponctuelle, ☐ régulière ou ☐ sur réquisition

13.Echangez-vous en face à face ou au téléphone autour de cas concrets de patients avec …

	Très souvent	*Souvent*	*De temps en temps*	*Rarement*
A - un / des médecins généralistes	☐	☐	☐	☐
B - un / des médecins spécialistes	☐	☐	☐	☐
C - un / des infirmiers	☐	☐	☐	☐
D - un / des pharmaciens	☐	☐	☐	☐
E - autre, qui ? :	☐	☐	☐	☐

14. Quel(s) professionnel(s) avez-vous le plus de difficultés à solliciter pour vos patients du fait de

(*plusieurs réponses possibles*)	*Un spécialiste en ville*	*Un spécialiste à l'hôpital*	*Un infirmier / paramédical*	*Un pharmacien*
a – … de l'éloignement	☐	☐	☐	☐
b – … du délai de RDV	☐	☐	☐	☐
c – … du dépassement d'honoraires	☐	☐	☐	☐
d – … de sa méconnaissance *(identité, compétence, coordonnées)*	☐	☐	☐	☐

B - Contenu de votre activité et caractéristique de votre patientèle

☛ Votre activité

1 - Avez-vous un Mode d'Exercice Particulier ? ☐ Oui ☐ Non

2 - Si oui lequel ? ....

3 - Avez-vous une activité spécialisée ? *(gynéco, pédiatrie, sport, gériatrie, etc.)* ☐ Oui ☐ Non

4 - Si oui laquelle ? ....

5 - Quelle part de votre temps de travail consacrez-vous à cette ou ces activités ? ....%

6 - Exercez-vous des activités d'enseignement ? *(université ou autre)* ☐ Oui ☐ Non

7 - Êtes-vous maître de stage ? ☐ Oui ☐ Non

15.Quelles sont les indemnités ou rémunérations annuelles de vos différentes activités en 2008 ?
(Relevé SNIR 2008)

- A-.Permanence des soins /gardes NBR [MAJF, MAJN, MAJU] .. .. *(en nombre)*
  Indemnités permanence téléphonique MNT [FRG] .. .. €
- B- Forfaits de coordination MNT [FC 0-9] .. .. €
- C- Aide pérenne à la transmission MNT [FFS] .. .. €
- D- Indemnités de déplacement NBR [ID + IF + IK] .. .. *(en nombre)*
  Dont frais de déplacement non remboursables MNT [FNR] .. .. €
- E- Formations diverses MNT [FF 0-9] + autres sources .. .. €

- F- Indemnités Maître de stage .. .. €
- G- Aides à l'installation *(régionale, Cnamts)* .. .. €
- H- Indemnités *(élu professionnel, autre activité professionnelle)* .. .. €
- G- Autres ? .... €

		Très souvent	Souvent	De temps en temps	Rarement
**8 -**	**Réalisez-vous ?** (*plusieurs réponses possibles*)				
	a – Un suivi gynécologique	☐	☐	☐	☐
	b - De la petite chirurgie (plâtre, points de suture, etc.)	☐	☐	☐	☐
	c - De la petite traumatologie	☐	☐	☐	☐
	d - Un test à l'effort	☐	☐	☐	☐
	e - Un électrocardiogramme	☐	☐	☐	☐
	e - De la prévention du tabac	☐	☐	☐	☐
	f - De la prévention de l'alcool	☐	☐	☐	☐
	g - De la prévention contre l'obésité	☐	☐	☐	☐
	h - Un suivi de grossesse	☐	☐	☐	☐
	i – une prise en charge de toxicomane	☐	☐	☐	☐
	j – Un suivi pédiatrique	☐	☐	☐	☐

9 - Participez-vous à un réseau médical de prise en charge? ☐ Oui ☐ Non

10 - Si oui, le ou lesquels ? : ....

11 - Combien de demi-journées travaillez-vous *en moyenne* par semaine ? .. .. demi-journées

12 - Combien d'heures travaillez-vous *en moyenne* par semaine ? .. .. .. heures

13 - Combien de semaines de vacances prenez-vous *en moyenne* par an ? .. .. semaines

14 - Combien de consultations avez-vous réalisé en 2008 ?
(RIAP 2008) .. ..

15 - Combien de visites avez-vous réalisé en 2008 ?
(RIAP 2008) .. ..

16 - Quel est le montant remboursable des actes que vous avez réalisés en 2008 ?
(RIAP 2008) .. .. €

☛ Votre patientèle

(RIAP 2008)

**17 - Nombre de patients dont vous êtes le « médecin traitant »**	**.. .. ..**
**18 - % de patients exonérés du TM**	**.. .. %**
**19 - % de bénéficiaires de la CMU**	**.. .. %**
**20 - % des actifs ayant eu des IJ**	**.. .. %**
**21 - % de patients de moins de 16 ans**	**.. .. %**
**22 - % de patients de 16 à 59 ans**	**.. .. %**
**23 - % de patients de 60 à 69 ans**	**.. .. %**
**24 - % de patients de 70 ans et plus**	**.. .. %**

C – Votre satisfaction et vos contraintes professionnelles

	Souvent	*De temps en temps*	*Rarement*	*Jamais*
**1 - Êtes-vous satisfait de votre activité professionnelle ?**	☐	☐	☐	☐
**2 - Vous sentez-vous libre de modifier votre agenda ?**	☐	☐	☐	☐
**3 - Vous sentez-vous contraint par les exigences de vos patients sur…**				
A- les horaires d'ouverture du cabinet	☐	☐	☐	☐
B- la durée de vos consultations	☐	☐	☐	☐
C - vos prescriptions médicamenteuses	☐	☐	☐	☐
D - vos prescriptions d'analyses et tests	☐	☐	☐	☐
E - vos prescriptions d'arrêts de travail	☐	☐	☐	☐
F - les orientations vers les spécialistes dans le cadre du parcours de soins	☐	☐	☐	☐
**4 - Considérez-vous que votre profession est gratifiante sur le plan humain**	☐	☐	☐	☐
**5 - Avez-vous un sentiment de *burn out* ?**	☐	☐	☐	☐
**6 - Êtes-vous confronté à des patients de plus en plus informés ?**			☐ Oui	☐ Non
**7 -** Si oui, considérez-vous que c'est une contrainte ?	☐	☐	☐	☐
**8 - Avez-vous envie de changer …**				
A - de spécialité	☐	☐	☐	☐
B - de lieu d'installation	☐	☐	☐	☐
C - de statut *(libéral / salarié)*	☐	☐	☐	☐
D - de mode d'installation *(individuel ⇄ groupe)*	☐	☐	☐	☐

9 - Êtes-vous satisfait de la rémunération de votre activité médicale libérale ? ☐ Oui ☐ Non

10 - Que représentent vos salaires éventuels (bruts) par rapport à vos revenus totaux ? .. .. %

11 - Vous sentez-vous contraints par le contrôle de l'assurance maladie ? ☐ Oui ☐ Non

12 - Si oui, est-ce que cela modifie votre pratique ? ☐ Oui ☐ Non

13 - Rencontrez-vous des difficultés particulières dues au profil de votre patientèle ? ☐ Oui ☐ Non

14 - Si oui, la ou lesquelles ? ……………………………………………………………………………………
………
………

15 - Avez-vous déjà ressenti un sentiment d'insécurité dans votre exercice ☐ Oui ☐ Non

D – Quelques questions supplémentaires

1.	**Sexe :**	☐ Homme	☐ Femme		
2.	**Age :**	.. .. ans			
3.	**Année d'installation :**	.. ..			
4.	**Avez-vous changé de mode d'installation dans les 5 dernières années** *(individuel ⇄ groupe)*		☐ Oui	☐ Non	
5.	**Département**	.. ..			
6.	**Dans une commune de … habitants**	☐ Moins de 10 000	☐ De 10 à 20 000	☐ De 20 à 100 000	☐ Plus de 100 000
7.	**Exercez-vous en Zone Franche Urbaine** *(ZFU)*		☐ Oui	☐ Non	
8.	**Exercez-vous en Zone de Revitalisation Rurale** *(ZRR)*		☐ Oui	☐ Non	
9.	**Avez-vous un ou des enfants à charge de moins de 15 ans**		☐ Oui	☐ Non	
10.	Si oui quel âge a le plus jeune		.. .. ans		
11.	**Comment percevez-vous votre santé ?**	Excellente ☐	Très bonne ☐	Bonne ☐	Médiocre ☐
12.	**Êtes-vous abonné à une revue professionnelle ?**		☐ Oui	☐ Non	
13.	**Suivez-vous des cycles de formation médicale continue**		☐ Oui	☐ Non	
14.	**Avez-vous déjà participé à une action d'Evaluation des Pratiques Professionnelles**		☐ Oui	☐ Non	

MERCI POUR VOTRE PARTICIPATION
