# Supplementary material for: What determines the income gap between French male and female GPs - the role of medical practices
Source: BMC Fam Pract. 2012 Sep 21;13:94. doi: 10.1186/1471-2296-13-94 (PMC3515453; doi:10.1186/1471-2296-13-94)
Supplement: Additional file 2 — Description of the available variables. [file 1471-2296-13-94-S2.docx]

**Additional file 2: Description of the available variables**

| ***Variables*** | | |
| --- | --- | --- |
| **Gross income (GI)** | | |
| ***GPs’ characteristics*** | | |
| **Years of experience**  *=Number of years in private practice* |  | **Teaching position** |
| **Number of years in private practice squared** |  |  |
| **Having a child under the age of 15** |  | **Sector 2**  *=1 if GPs have the option of charging higher fees* |
| **Reported being in good health** |  | **Rural region** |
| =1 if GPs declare having excellent, very good or good health |  | *=1 if GPs work in communes counting less than 10,000 inhabitants in French territorial division of Haute Garonne that benefit from the influence of Toulouse (4th largest French city)* |
| **Age** |  |  |
| ***Workload*** | | |
| **Salaried activity (%)** |  | **Number of working days per week** |
| *represented the percentage of salaries in the private medical practice turnover* |  | **Number of hours worked per day** |
| **Number of consultations/visits per year** |  | **Number of vacations** *(Number of weeks off)* |
| ***Type of practice*** | | |
| **Participating in on-going care** |  | **Group practice** |
| **Percentage of visits in the number of consultations/visits per year** |  |  |
| **Have consultations by appointment** |  | **Specialised practice** |
| *=1 if GPs practice only consultations by appointment* |  | *=1 if GPs practice certain specific activity (acupuncture, homeopathy, dietetic, etc.) more than 30% of his time.* |
| **Frequently perform obstetric follow-ups** |  | **Frequently perform gynaecologic follow-ups** |
| *=1 if GPs declare performing obstetric follow-ups frequently or less frequently* |  | *=1 if GPs declare performing gynaecologic follow-ups frequently or less frequently* |
| **Frequently perform paediatric follow-ups** |  | **Frequently perform electrocardiograms** |
| *=1 if GPs declare performing paediatric follow-ups frequently or less frequently* |  | *=1 if GPs declare performing electrocardiograms frequently or less frequently* |
| **Frequently perform traumatology** |  | **Frequently perform minor surgeries** |
| *=1 if GPs declare performing traumatology frequently or less frequently* |  | *=1 if GPs declare performing minor surgeries frequently or less frequently* |
| ***Type of patients*** | | |
| **Have many children in their patients list (aged under 16)** |  | **Have many elderly in their patients list (aged over 65)** |
| *=1 if the GP has a percentage of children under 16 higher than average* |  | *=1 if the GP has a percentage of elderly higher than average* |
